# Surgical outcomes in gastroenterological surgery in Japan: Report of National Clinical database 2011–2016

**DOI:** 10.1002/ags3.12052

**Published:** 2017-11-23

**Authors:** Yoshihiro Kakeji, Arata Takahashi, Harushi Udagawa, Michiaki Unno, Itaru Endo, Chikara Kunisaki, Akinobu Taketomi, Akira Tangoku, Tadahiko Masaki, Shigeru Marubashi, Kazuhiro Yoshida, Mitsukazu Gotoh, Hiroyuki Konno, Hiroaki Miyata, Yasuyuki Seto, National Clinical Database

**Affiliations:** ^1^ The Japanese Society of Gastroenterological Surgery Tokyo Japan; ^2^ Department of Health Policy and Management School of Medicine Keio University Kobe Japan; ^3^ Department of Healthcare Quality Assessment, Graduate School of Medicine The University of Tokyo Tokyo Japan

**Keywords:** gastroenterological surgery, National Clinical Database, surgical outcome

## Abstract

The National Clinical Database (NCD) of Japan started its registration in 2011 and over 9 000 000 cases from more than 5000 facilities were registered over a 6‐year period. This is the report of NCD based upon gastrointestinal surgery information in excess of 3 200 000 cases from 2011 to 2016 adding data of complications. About 70% of all gastrointestinal surgeries were carried out at certified institutions, and the percentage of surgeries done at certified institutions was particularly high for the esophagus (92.4% in 2016), liver (88.4%), pancreas (89.8%), and spleen (86.8%). The percentage of anesthesiologist participation was more than 90% for almost all organs, except 85.7% for the rectum and anus. Approximately, more than two‐thirds of the surgeries were carried out with the participation of a board‐certified surgeon. Although patients have been getting older, mortalities have not been increasing. There were differences in the incidence of complications according to organ site and procedure. Remarkably, mortality rates of low anterior resection were very low, and those of hepatectomy and acute diffuse peritonitis surgery have been gradually decreasing. Although the complication rates were gradually increasing for esophagectomy or pancreaticoduodenectomy, the mortality rates for these procedures were decreasing. Nationwide, this database is expected to ensure the quality of the board‐certification system and surgical outcomes in gastroenterological surgery.

## 1 INTRODUCTION

The National Clinical Database (NCD) was founded in 2010 as the parent body of the database system linked to the board‐certification system.^1^ The NCD database project, which started recordkeeping in January 2011, covers records of ≥95% of the surgeries carried out by regular surgeons in Japan.^2^ Almost 5000 facilities have enrolled and over 9 100 000 cases have been registered as of the end of December 2016.

In the gastrointestinal surgery section, all surgical cases are registered and require detailed input items for eight procedures representing the performance of surgery in each specialty (esophagectomy, distal gastrectomy, total gastrectomy, right hemicolectomy, low anterior resection, hepatectomy, pancreatoduodenectomy, and surgery for acute diffuse peritonitis). Risk models of mortality for each procedure were created using approximately 120 000 cases registered in 2011, and each model has been accepted and published in peer‐reviewed journals.^3^, ^4^, ^5^, ^6^, ^7^, ^8^, ^9^, ^10^ All reports were the first‐risk stratification studies, based on a Japanese nationwide Web‐based database. Mortality rates were almost satisfactory compared to those in the Western world. In case of esophagectomy, risk models may not be markedly influenced by choice of open or laparoscopic esophagectomy.^3^ The 30‐day mortality may underestimate the true risk for death, and operative mortality is recommended as a standard outcome measure after colorectal surgery.^7^ As for acute diffuse peritonitis, 38.7% of the 8482 patients were admitted to a hospital by direct ambulance transport.^10^ Based on these studies, we can use a real‐time feedback system, which includes a risk calculator for the mortality (predicted postoperative 30‐day mortality and operative mortality) of preoperative patients and performance reports of each participating hospital.^11^ The latter shows each facility's severity‐adjusted clinical performance (benchmark) in comparison with the national data and the risk‐adjusted cumulative expected–observed death. Better or worse outcomes can be detected by the monitoring report. Furthermore, we are proceeding with papers on complications related to each of the eight operative methods for the evaluation of medical standards using data from 2011 and 2012.^12^, ^13^, ^14^, ^15^, ^16^, ^17^ To assure collection of high‐quality data, the Japanese Society of Gastroenterological Surgery (JSGS) have started data verification activities for a gastroenterological session in NCD in 2016.

Following up on the Annual Report 2011–2014,^18^, ^19^, ^20^ we herein report the NCD 2011–2016 based upon gastrointestinal surgery information in 3 215 977 cases of surgery carried out and recorded from 2011 to 2016 adding the data of complications. We would be satisfied if this report aided in the improvement of gastrointestinal surgery treatment in Japan.

## 2 SUBJECTS AND METHODS

Subjects were surgical data recorded in the NCD, which were stipulated by the “Training Curriculum for Board‐Certified Surgeons in Gastroenterology”, using the “New classification of surgical difficulty”. The board‐certification system of the Japanese Society of Gastroenterological Surgery (JSGS) consists of board‐certified training institutions and board‐certified surgeons in gastroenterological surgery.^21^ Requirements for board‐certified training institutions are 600 or more gastroenterological operations determined by the certified committee (more than 120 of which are essential major surgery) in the last 3 years. Board‐certified surgeons are also required to carry out 450 or more gastroenterological operations and gastroenterological surgical training for more than 5 years according to the training curriculum in a board‐certified training institution authorized by the JSGS. We targeted data from 2011 to 2016, adding data of complications to data already reported in the Annual Report 2011–2012, 2013, and 2014 on 115 gastrointestinal surgical procedures. Complications included surgical site infection (SSI), wound dehiscence, anastomotic leakage, pancreatic fistula, bile leakage, pneumonia, unplanned intubation, pulmonary embolism, ventilator‐assisted respiration longer than 48 hours, progressive renal insufficiency, acute renal failure, urinary tract infection, cerebrovascular accident with neurological deficit, coma longer than 24 hours, peripheral nerve injury, cardiac arrest requiring cardiopulmonary resuscitation, myocardial infarction, bleeding complications defined by transfusions in excess of 1 unit of blood, deep venous thrombosis, and sepsis. Postoperative complications were categorized into six grades according to the Clavien‐Dindo (C‐D) classification.^22^, ^23^ In this study, grade III (complications requiring intervention) or higher complications were defined as severe complications. Furthermore, we separated and studied the operative methods from among these 115 procedures that we deemed important in terms of medical standards as the eight main operative methods.

Here we clarified the number of surgical cases and the mortality rates related to the 115 selected gastrointestinal operative procedures. We also clarified the changes over time in the number of surgical cases and mortality rates related to the eight main operative procedures from 2011 to 2016. We also comparatively studied patient sex, age groups, institution groups, and percentage contribution of certified surgeons related to the eight main operative procedures.

The following points need to be considered in the interpretation of the data reported here. (i) As a maximum of eight operative procedures can be recorded for each case in the NCD, the total number of surgeries in “Results of the 115 gastrointestinal surgical procedures for board‐certification system” is not the actual total number of surgical cases. (ii) Cases with errors in patient age, sex, and postoperative 30‐day status were excluded. (iii) Cases in which several operative methods were carried out simultaneously were tallied for all operative methods. (iv) Postoperative 30‐day mortality included all cases of mortality within 30 days after surgery regardless of pre‐ or post‐discharge status. Calculation of operative mortality included all patients who died during the index hospitalization, including hospital stays of up to 90 days, and any patient who died after hospital discharge within 30 days of the operation date.

## 3 RESULTS

### 3.1 The 115 selected gastrointestinal operative procedures in the “Training Curriculum for Board‐Certified Surgeons in Gastroenterology”

The total number of cases represented by the 115 selected gastrointestinal surgical procedures recorded in the NCD between 1 January 2011 and 31 December 2016 was 3 215 977. Based on organ involvement, 51 883 cases involved the esophagus (1.6%); 439 540 cases the stomach and duodenum (13.7%); 1 174 168 cases the small intestine and colon (36.5%); 303 957 cases the rectum and anus (9.5%); 155 065 cases the liver (4.8%); 756 526 cases the gall bladder (23.5%); 98 365 cases the pancreas (3.1%); 23 271 cases the spleen (0.7%); and 213 202 cases other organs (6.6%) (Table 1). The increase of cases especially with malignant colorectal diseases was remarkable. The male : female ratio was approximately 6:4 overall, and there were some variations according to organs. Year by year, older patients have been increasing, especially for the stomach and duodenum, small intestine and colon, and rectum and anus (Table 1).

**Table 1:** Annual changes of surgeries by sex, age group, and organ for the 115 selected gastrointestinal operative procedures in the training curriculum for board‐certified surgeons in gastroenterology

Organ	Year	No. surgeries	Percentage by sex		Percentage according to age group (years)					
			Male	Female	<60	60 to <65	65 to <70	70 to <75	75 to <80	≥80
Esophagus	2011	7246	81.8	18.2	22.5	19.6	21.1	18.7	12.0	6.0
	2012	8819	82.2	17.8	22.1	19.7	20.0	19.5	12.9	6.0
	2013	8642	81.5	18.5	20.8	17.5	21.0	20.6	13.2	6.9
	2014	9021	81.5	18.4	20.8	16.5	21.4	20.9	13.8	6.6
	2015	8943	80.8	19.2	19.6	15.3	22.4	22.5	13.1	7.1
	2016	9212	79.6	20.4	20.1	14.4	22.9	20.5	14.5	7.5
Stomach and duodenum	2011	66 740	68.0	32.0	20.1	14.4	14.0	17.1	16.4	18.0
	2012	76 186	68.3	31.7	18.9	14.4	14.5	17.1	16.4	18.6
	2013	75 583	67.9	32.1	18.6	13.1	15.5	17.2	16.9	18.7
	2014	74 920	67.6	32.4	17.9	12.1	16.0	17.8	16.7	19.5
	2015	73 877	67.8	32.2	17.4	11.1	17.1	17.8	16.6	19.9
	2016	72 234	67.8	32.2	17.0	10.2	18.1	17.1	16.6	21.0
Small intestine and colon	2011	151 143	56.7	43.3	37.4	10.9	10.5	12.1	12.2	16.9
	2012	184 810	56.7	43.3	36.4	10.7	10.7	12.2	12.5	17.4
	2013	198 677	56.9	43.1	35.6	10.1	11.3	12.7	12.4	17.8
	2014	206 857	56.9	43.1	34.7	9.4	12.0	13.1	12.4	18.4
	2015	214 453	57.1	42.9	34.0	8.9	12.9	13.1	12.3	18.7
	2016	218 228	57.3	42.7	33.7	8.4	13.6	12.5	12.4	19.3
Rectum and anus	2011	41 061	59.1	40.9	22.0	16.1	14.6	15.4	14.2	17.7
	2012	49 704	58.3	41.7	22.3	14.8	14.6	15.5	14.3	18.5
	2013	49 980	58.0	42.0	20.9	13.9	15.2	16.1	14.6	19.3
	2014	51 454	58.3	41.7	20.4	13.1	16.0	16.4	14.2	19.9
	2015	56 092	57.8	42.2	22.3	11.8	16.7	15.7	14.0	19.4
	2016	55 666	57.3	42.7	22.0	11.1	17.9	15.0	13.6	20.4
Liver	2011	22 855	67.3	32.7	22.2	16.5	16.3	18.7	17.2	9.2
	2012	26 288	66.3	33.7	22.1	15.7	16.7	18.0	17.4	10.2
	2013	25 814	66.1	33.9	21.3	14.6	17.6	18.7	17.3	10.5
	2014	26 518	66.3	33.7	21.5	13.7	18.1	19.8	16.6	10.3
	2015	26 378	65.7	34.3	20.8	12.8	18.9	19.4	16.5	11.5
	2016	27 212	66.4	33.6	20.3	11.5	20.5	18.6	17.0	12.1
Gall bladder	2011	103 183	54.5	45.4	34.3	14.0	12.2	13.8	12.8	13.0
	2012	122 513	55.2	44.8	32.9	13.8	12.4	13.9	13.2	13.8
	2013	129 162	55.3	44.7	32.6	12.9	13.0	14.2	13.2	14.0
	2014	131 182	55.6	44.4	32.1	11.8	13.9	14.5	13.2	14.5
	2015	133 126	55.6	44.4	32.0	11.2	15.0	14.1	13.0	14.8
	2016	137 360	55.4	44.6	32.6	10.6	15.5	13.1	12.9	15.3
Pancreas	2011	13 477	59.9	40.1	20.0	15.6	16.9	19.7	17.7	10.2
	2012	15 550	60.0	40.0	19.8	15.2	17.0	19.5	18.2	10.3
	2013	16 380	59.7	40.3	19.1	13.6	18.0	20.7	17.7	10.9
	2014	17 313	59.5	40.5	18.4	12.4	19.0	21.0	18.2	11.1
	2015	17 407	59.1	40.9	18.2	11.3	19.4	21.6	18.1	11.4
	2016	18 238	58.9	41.1	18.2	10.4	19.9	20.4	19.0	12.2
Spleen	2011	3609	61.3	38.7	35.3	15.6	14.7	14.8	11.9	7.8
	2012	4142	61.4	38.6	32.9	16.3	15.0	15.1	12.9	7.8
	2013	4509	61.8	38.2	30.8	14.9	15.9	16.5	13.1	8.7
	2014	4272	61.8	38.2	29.9	13.0	17.3	17.0	13.8	9.1
	2015	3568	60.4	39.6	29.7	11.4	17.3	16.6	14.1	10.8
	2016	3171	57.3	42.7	31.9	11.7	17.7	15.7	12.5	10.5
Others	2011	23 218	55.0	45.0	32.0	11.9	11.3	13.3	13.8	17.6
	2012	28 779	55.4	44.6	31.1	11.7	11.7	13.8	13.7	18.0
	2013	36 363	53.1	46.9	28.3	10.9	12.7	14.1	14.8	19.1
	2014	39 854	53.7	46.3	28.1	10.1	13.1	14.5	14.4	19.8
	2015	41 465	53.2	46.8	27.4	9.4	14.0	14.5	14.2	20.6
	2016	43 523	54.0	46.0	27.5	9.2	14.6	13.5	14.0	21.2

**Figure 1:**
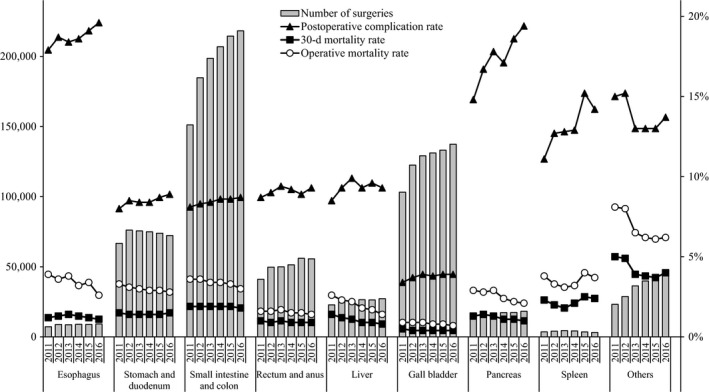
Annual changes of number of surgeries, 30‐day mortality, operative mortality, and complications: Analysis of 115 surgical procedures. Postoperative complication rate: the rate of Clavien‐Dindo (C‐D) classification grade III (complications requiring intervention) or higher complications.

In terms of the institution groups in which the surgeries were carried out, approximately 70% of all surgeries were done at certified institutions, and the percentage of surgeries carried out at certified institutions was particularly high for the esophagus (92.4% in 2016), liver (88.4%), pancreas (89.8%), and spleen (86.8%) (Table 2). The percentage of anesthesiologist participation was more than 90% for almost all organs, except 85.7% for the rectum and anus. Approximately more than two‐thirds of the surgeries were carried out with the participation of a board‐certified surgeon. The percentage of certified surgeons that were operators was high for the esophagus (70.0% in 2016), liver (59.6%), and pancreas (62.4%). The total number of recorded surgeries increased each year (Figure 1). Postoperative complications, 30‐day mortality rates, and operative mortality rates are shown in Table 3. Complication rates were comparatively higher for the esophagus and the pancreas; however, the mortality rates for these organ procedures were not so high. Figure 1 shows number of surgeries, mortality rates, and complications of the 115 gastrointestinal surgical procedures according to organ involvement. Tables 4, 5, 6, 7, 8, 9, 10, 11, 12 show the number of surgeries using each of the 115 gastrointestinal surgical procedures, according to recording year and organ.

**Table 2:** Institution and anesthesiologist and specialist participation rates by organ for the 115 selected gastrointestinal operative procedures

Organ	Year	No. surgeries	Percentage by institution group			Anesthesiologist participation (%)	Board‐certified surgeon participation (%)	Medical practitioners (%)	
			Certified institution	Related institution	Other			Board‐certified surgeons	Non‐board‐certified surgeons
Esophagus	2011	7246	93.5	5.9	0.6	97.0	87.0	62.8	37.2
	2012	8819	78.1	5.9	16.0	97.4	87.0	62.7	37.3
	2013	8642	90.6	7.1	2.4	97.3	88.4	64.4	35.6
	2014	9021	91.1	6.1	2.8	97.9	90.1	67.6	32.4
	2015	8943	91.5	6.0	2.5	97.9	91.1	69.4	30.6
	2016	9212	92.4	5.0	2.6	98.2	91.2	70.0	30.0
Stomach and duodenum	2011	66 740	80.2	17.3	2.6	92.8	69.3	35.1	64.9
	2012	76 186	63.5	15.6	20.9	93.5	70.3	35.6	64.4
	2013	75 583	76.3	19.3	4.4	93.3	73.5	37.7	62.3
	2014	74 920	77.0	18.2	4.8	93.6	75.9	39.2	60.8
	2015	73 877	77.1	18.3	4.6	93.9	76.1	39.2	60.8
	2016	72 234	79.6	16.1	4.3	94.6	78.7	41.0	59.0
Small intestine and colon	2011	151 143	76.8	20.2	2.9	88.1	59.2	25.1	74.9
	2012	184 810	60.6	18.2	21.2	88.9	59.9	25.4	74.6
	2013	198 677	72.6	22.2	5.2	89.6	62.7	26.6	73.4
	2014	206 857	73.0	21.4	5.6	90.8	65.4	28.1	71.9
	2015	214 453	73.8	20.7	5.5	91.6	66.3	28.5	71.5
	2016	218 228	75.6	19.0	5.5	92.4	68.1	29.5	70.5
Rectum and anus	2011	41 061	76.9	19.0	4.1	86.3	68.3	36.9	63.1
	2012	49 704	60.4	18.2	21.4	85.7	68.6	37.6	62.4
	2013	49 980	72.9	21.7	5.4	87.3	71.2	39.4	60.6
	2014	51 454	73.5	20.9	5.6	87.9	73.7	41.6	58.4
	2015	56 092	72.5	20.8	6.7	84.9	73.5	41.5	58.5
	2016	55 666	74.1	19.4	6.6	85.7	74.7	42.1	57.9
Liver	2011	22 855	89.3	9.7	1.1	95.6	85.2	55.2	44.8
	2012	26 288	74.2	9.2	16.7	95.4	85.7	57.4	42.6
	2013	25 814	86.3	10.7	2.9	96.3	87.5	57.1	42.9
	2014	26 518	86.3	10.0	3.7	96.4	89.0	59.6	40.4
	2015	26 378	87.3	9.5	3.2	96.6	89.1	59.1	40.9
	2016	27 212	88.4	8.8	2.9	96.8	90.0	59.6	40.4
Gall bladder	2011	103 183	73.9	22.5	3.6	91.8	61.9	26.4	73.6
	2012	122 513	57.5	19.6	22.9	92.1	62.8	26.3	73.7
	2013	129 162	69.9	24.1	5.9	92.2	65.4	27.3	72.7
	2014	131 182	70.3	23.3	6.4	92.3	67.4	28.1	71.9
	2015	133 126	70.8	22.8	6.4	92.9	68.4	28.1	71.9
	2016	137 360	72.4	21.3	6.3	93.5	69.4	28.9	71.1
Pancreas	2011	13 477	88.1	10.8	1.2	95.8	85.2	57.7	42.3
	2012	15 550	72.8	8.7	18.5	96.3	86.5	59.9	40.1
	2013	16 380	86.5	11.0	2.4	95.9	87.6	60.2	39.8
	2014	17 313	86.9	9.9	3.3	96.2	89.1	61.3	38.7
	2015	17 407	88.4	9.1	2.4	96.4	90.3	61.6	38.4
	2016	18 238	89.8	8.0	2.3	96.8	91.1	62.4	37.6
Spleen	2011	3609	87.0	11.6	1.4	94.6	75.2	44.9	55.1
	2012	4142	70.5	9.5	20.0	81.7	75.8	44.4	55.6
	2013	4509	83.2	13.8	3.0	95.2	75.4	43.3	56.7
	2014	4272	85.4	11.5	3.1	94.6	77.5	45.2	54.8
	2015	3568	85.6	12.3	2.1	94.8	78.9	45.5	54.5
	2016	3171	86.8	10.1	3.1	95.7	80.5	48.0	52.0
Others	2011	23 218	80.2	17.0	2.8	90.3	60.4	27.2	72.8
	2012	28 779	65.7	15.2	19.1	91.0	61.1	27.6	72.4
	2013	36 363	76.1	19.3	4.6	91.5	63.4	28.5	71.5
	2014	39 854	76.6	18.2	5.1	91.9	64.9	29.7	70.3
	2015	41 465	78.0	17.2	4.8	92.4	65.6	29.4	70.6
	2016	43 523	79.4	15.8	4.8	92.7	67.3	30.3	69.7

**Table 3:** No. surgeries and mortality rates according to organ treated using the 115 selected gastrointestinal operative procedures

Organ	Year	No. surgeries	No. postoperative complications^a^/rate (%)	No. 30‐day mortalities/rate (%)	No. operative mortalities/rate (%)
Esophagus	2011	7246	1294/17.9	87/1.2	279/3.9
	2012	8819	1653/18.7	117/1.3	315/3.6
	2013	8642	1593/18.4	121/1.4	327/3.8
	2014	9021	1679/18.6	115/1.3	289/3.2
	2015	8943	1709/19.1	103/1.2	304/3.4
	2016	9212	1805/19.6	100/1.1	238/2.6
Stomach and duodenum	2011	66 740	5354/8.0	992/1.5	2183/3.3
	2012	76 186	6447/8.5	1085/1.4	2381/3.1
	2013	75 583	6380/8.4	1059/1.4	2269/3.0
	2014	74 920	6328/8.4	1064/1.4	2174/2.9
	2015	73 877	6418/8.7	1007/1.4	2110/2.9
	2016	72 234	6413/8.9	1066/1.5	2016/2.8
Small intestine and colon	2011	151 143	12 184/8.1	2943/1.9	5390/3.6
	2012	184 810	15 395/8.3	3564/1.9	6583/3.6
	2013	198 677	16 709/8.4	3723/1.9	6803/3.4
	2014	206 857	17 776/8.6	3822/1.9	6961/3.4
	2015	214 453	18 372/8.6	4019/1.9	7092/3.3
	2016	218 228	19 020/8.7	3933/1.8	6621/3.0
Rectum and anus	2011	41 061	3584/8.7	395/1.0	676/1.6
	2012	49 704	4488/9.0	462/0.9	802/1.6
	2013	49 980	4684/9.4	517/1.0	858/1.7
	2014	51 454	4711/9.2	449/0.9	792/1.5
	2015	56 092	4986/8.9	519/0.9	824/1.5
	2016	55 666	5194/9.3	503/0.9	766/1.4
Liver	2011	22 855	1933/8.5	309/1.4	590/2.6
	2012	26 288	2454/9.3	310/1.2	605/2.3
	2013	25 814	2549/9.9	275/1.1	575/2.2
	2014	26 518	2466/9.3	246/0.9	481/1.8
	2015	26 378	2537/9.6	234/0.9	451/1.7
	2016	27 212	2543/9.3	222/0.8	382/1.4
Gall bladder	2011	103 183	3473/3.4	483/0.5	946/0.9
	2012	122 513	4587/3.7	531/0.4	1082/0.9
	2013	129 162	4982/3.9	546/0.4	1130/0.9
	2014	131 182	5020/3.8	569/0.4	1097/0.8
	2015	133 126	5231/3.9	541/0.4	1036/0.8
	2016	137 360	5320/3.9	559/0.4	980/0.7
Pancreas	2011	13 477	1994/14.8	175/1.3	386/2.9
	2012	15 550	2595/16.7	213/1.4	437/2.8
	2013	16 380	2917/17.8	211/1.3	482/2.9
	2014	17 313	2966/17.1	195/1.1	423/2.4
	2015	17 407	3229/18.6	185/1.1	379/2.2
	2016	18 238	3543/19.4	185/1.0	390/2.1
Spleen	2011	3609	400/11.1	83/2.3	137/3.8
	2012	4142	528/12.7	84/2.0	138/3.3
	2013	4509	575/12.8	79/1.8	139/3.1
	2014	4272	549/12.9	88/2.1	137/3.2
	2015	3568	543/15.2	88/2.5	144/4.0
	2016	3171	449/14.2	76/2.4	117/3.7
Others	2011	23 218	3494/15.0	1163/5.0	1887/8.1
	2012	28 779	4388/15.2	1399/4.9	2293/8.0
	2013	36 363	4712/13.0	1401/3.9	2346/6.5
	2014	39 854	5176/13.0	1521/3.8	2489/6.2
	2015	41 465	5380/13.0	1541/3.7	2545/6.1
	2016	43 523	5975/13.7	1760/4.0	2684/6.2

a Complications were defined by Clavien‐Dindo grade IIIa‐V.

**Table 4:** Annual changes in the number of surgeries according to the 115 selected gastrointestinal operative procedures (esophagus)

Organ	Degree of difficulty	Procedure	No. surgeries					
			2011	2012	2013	2014	2015	2016
Esophagus	Low	Cervical periesophageal abscess drainage	23	27	34	42	37	43
	Med	Esophageal suture (perforation, injury)	156	204	198	185	199	215
	Med	Thoracic periesophageal abscess drainage	22	23	18	27	27	21
	Med	Esophageal foreign body extraction	19	21	26	25	30	32
	Med	Esophageal diverticulum resection	27	32	35	48	41	34
	Med	Benign esophageal tumor removal	61	69	66	68	52	64
	Med	Esophageal resection (removal only)	388	506	580	570	571	721
	Med	Esophageal reconstruction: reconstruction only (gastric tube reconstruction)	699	844	888	799	848	772
	Med	Esophageal fistula construction	97	106	128	126	125	162
	Med	Esophagocardioplasty	321	418	392	398	362	365
	Med	Achalasia surgery	77	109	84	118	101	210
	High	Esophagectomy	4916	5946	5694	6091	6060	6041
	High	Esophageal reconstruction: reconstruction only (colon reconstruction)	65	56	63	77	51	40
	High	Esophageal bypass	93	110	137	143	152	130
	High	Bronchoesophageal fistula surgery	6	5	9	12	7	13
	High	Secondary esophageal reconstruction	276	343	290	292	280	349

**Table 5:** Annual changes in the number of surgeries according to the 115 selected gastrointestinal operative procedures (stomach and duodenum)

Organ	Degree of difficulty	Procedure	No. surgeries					
			2011	2012	2013	2014	2015	2016
Stomach and duodenum	Low	Gastrostomy and suture gastrorrhaphy	52	69	74	66	65	77
	Low	Diverticulum, polypectomy (excluding endoscopic resection)	156	186	231	247	226	202
	Low	Truncal vagotomy	3	6	6	2	6	3
	Low	Gastroenterostomy (including duodenal jejunostomy)	4651	5330	5571	5893	5636	5633
	Low	Gastric fistula construction (excluding PEG)	1717	1698	1633	1722	1790	1748
	Low	Gastric pyloroplasty	116	129	115	126	100	69
	Low	Gastric volvulus (volvulus) surgery and rectopexy	40	38	39	0	47	42
	Low	Gastric suture (including gastric suture for gastric rupture, suture closure for gastroduodenal perforation, omental implantation and omental transposition)	4707	5738	5669	5837	5858	6164
	Low	Local gastrectomy (including wedge resection)	2466	3108	3233	3354	3625	3766
	Med	Gastrectomy (including distal gastrectomy, pylorus preserving gastrectomy and segmental [transverse] gastrectomy)	34 160	38 750	39 957	38 584	37 819	36 852
	Med	Selective vagotomy	8	8	10	7	6	4
	High	Total gastrectomy (including fundusectomy)	18 652	21 122	19 035	19 071	18 695	17 670
	High	Left upper abdominal exenteration	12	4	10	11	4	4

**Table 6:** Annual changes in the number of surgeries according to the 115 selected gastrointestinal operative procedures (small intestine and colon)

Organ	Degree of difficulty	Procedure	No. surgeries					
			2011	2012	2013	2014	2015	2016
Small intestine and colon	Low	Enterotomy and enterorrhaphy	2982	3505	4025	4362	4412	4311
	Low	Disinvagination (invasive)	172	250	234	239	209	242
	Low	Partial enterectomy (benign)	5792	7602	8564	8938	9449	9591
	Low	Ileocecal resection (benign)	3238	4104	4313	4472	4523	4675
	Low	Partial colectomy and sigmoid colectomy (benign)	4946	6239	6626	7358	7583	7971
	Low	Appendectomy	43 437	51 316	54 421	54 319	54 897	55 168
	Low	Enterostomy and closure (without enterectomy)	15 192	19 371	21 600	23 425	24 666	25 458
	Med	Enterectomy (malignant)	2448	2703	3016	3082	3320	3360
	Med	Ileocecal resection (malignant)	5492	9274	10 327	11 368	12 224	12 872
	Med	Partial colectomy and sigmoid colectomy (malignant)	25 034	29 863	31 495	32 092	33 518	33 936
	Med	Right hemicolectomy	17 890	21 034	21 814	22 446	22 850	22 829
	Med	Left hemicolectomy	5241	5347	5644	5763	6119	6178
	Med	Total colectomy	2846	3131	1892	1701	1752	1735
	Med	Intestinal obstruction surgery (with bowel resection)	5117	6496	7412	7775	7912	7898
	Med	Enterostomy and closure (with enterectomy)	11 008	14 162	16 853	19 049	20 520	21 525
	High	Proctocolectomy and ileoanal (canal) anastomosis	308	413	441	468	499	479

**Table 7:** Annual changes in the number of surgeries according to the 115 selected gastrointestinal operative procedures (rectum and anus)

Organ	Degree of difficulty	Procedure	No. surgeries					
			2011	2012	2013	2014	2015	2016
Rectum and anus	Low	Transanal rectal tumor removal	2483	3300	1657	1513	3690	3651
	Low	Proctocele surgery (transanal)	1802	2461	2488	2602	2773	2805
	Med	Rectectomy (benign)	300	386	2196	2060	1914	1688
	Med	High anterior resection	7053	8920	8985	9496	9934	10 477
	Med	Hartmann's procedure	3562	4614	4865	5194	5650	5755
	Med	Proctocele surgery (abdominoperineal)	659	996	1119	1181	1411	1538
	Med	Malignant anorectal tumor excision (transanal)	1517	1037	898	864	821	778
	Med	Anal sphincteroplasty (by tissue replacement)	969	1378	1721	1718	2132	2045
	High	Rectectomy (malignant)	5308	5828	4474	4531	4825	5096
	High	Low anterior resection	16 984	20 321	21 096	21 861	22 493	21 387
	High	Pelvic evisceration	359	389	412	374	385	402
	High	Anorectal malignant tumor excision (posterior approach)	65	74	69	60	64	44

**Table 8:** Annual changes in the number of surgeries according to the 115 selected gastrointestinal operative procedures (liver)

Organ	Degree of difficulty	Procedure	No. surgeries					
			2011	2012	2013	2014	2015	2016
Liver	Low	Hepatorrhaphy	172	202	161	196	147	161
	Low	Liver abscess drainage (excluding percutaneous procedures)	42	47	54	44	59	55
	Low	Hepatic cyst resection. Suture. Drainage	425	535	606	695	695	741
	Low	Partial hepatectomy	9431	10 919	10 708	11 598	12 063	12 604
	Low	Liver biopsy (excluding percutaneous procedures)	122	264	176	165	175	126
	Low	Liver coagulonecrotic therapy (excluding percutaneous procedures)	1958	2122	1083	1069	939	854
	Med	Lateral segmentectomy of the liver	1390	1632	1773	1807	1666	1704
	Med	Esophageal and gastric varix surgery	94	109	67	61	46	67
	High	Hepatectomy (segmented or more; excluding lateral segments)	7434	8239	7937	7666	7439	7610
	High	Systematic subsegmentectomy	996	1353	2374	2257	2221	2367
	High	Liver transplant	692	775	757	848	790	800
	High	Hepatopancreatoduodenectomy	99	91	118	112	138	123

**Table 9:** Annual changes in the number of surgeries according to the 115 selected gastrointestinal operative procedures (gall bladder)

Organ	Degree of difficulty	Procedure	No. surgeries					
			2011	2012	2013	2014	2015	2016
Gall bladder	Low	Cholangiotomy	142	163	174	139	141	132
	Low	Cysticolithectomy	1094	1093	750	641	611	571
	Low	Cholecystectomy	93 665	112 048	119 455	122 026	124 267	128 809
	Low	External cholecystostomy	104	119	127	124	109	146
	Low	Cystoenteric anastomosis	70	73	61	61	67	59
	Med	Cysticolithectomy	3682	4117	3880	3574	3342	3057
	Med	Biliary tract reconstruction	150	162	265	315	362	347
	Med	Biliary bypass	1594	1751	1765	1686	1613	1490
	Med	Cholangioplasty	201	180	192	168	156	176
	Med	Duodenal papilloplasty	66	68	50	33	31	37
	Med	Choledochal dilatation	217	240	254	242	248	291
	Med	Biliary fistula closure	43	42	42	37	40	34
	High	Malignant gallbladder tumor surgery (excluding simple cholecystectomy)	869	1013	929	963	969	948
	High	Malignant bile duct tumor surgery	1268	1426	1202	1153	1155	1245
	High	Biliary atresia surgery	18	18	16	20	15	18

**Table 10:** Annual changes in the number of surgeries according to the 115 selected gastrointestinal operative procedures (pancreas)

Organ	Degree of difficulty	Procedure	No. surgeries					
			2011	2012	2013	2014	2015	2016
Pancreas	Low	External pancreatic cyst drainage	29	27	13	21	8	13
	Low	External pancreatic duct drainage	17	20	26	28	22	34
	Med	Pancreatorrhaphy	22	17	21	34	27	17
	Med	Partial pancreatic resection	126	148	202	182	165	177
	Med	Distal pancreatectomy (benign)	1018	1398	1372	1557	1477	1536
	Med	Pancreatoenteric anastomosis	81	71	59	49	44	39
	Med	Pancreatic (duct) anastomosis	223	295	309	388	280	269
	Med	Acute pancreatitis surgery	94	117	104	103	90	132
	Med	Pancreatolithiasis surgery	17	17	14	35	31	29
	Med	Plexus pancreaticus capitalis resection	1	1	2	0	1	1
	High	Pancreaticoduodenectomy	8305	9329	10 068	10 400	10 576	11 028
	High	Distal pancreatectomy (malignant)	2861	3344	3483	3750	3930	4173
	High	Total pancreatectomy	348	408	423	496	503	545
	High	Duodenum preserving pancreas head resection	201	193	111	85	63	49
	High	Segmental pancreatic resection	131	163	138	165	162	169
	High	Distal pancreatectomy	3	2	35	20	28	27

**Table 11:** Annual changes in the number of surgeries according to the 115 selected gastrointestinal operative procedures (spleen)

Organ	Degree of difficulty	Procedure	No. surgeries					
			2011	2012	2013	2014	2015	2016
Spleen	Low	Splenorrhaphy	22	35	26	24	17	30
	Med	Splenectomy	3564	4063	4457	4215	3525	3117
	Med	Partial splenic resection	23	44	26	33	26	24

**Table 12:** Annual changes in the number of surgeries according to the 115 selected gastrointestinal operative procedures (other)

Organ	Degree of difficulty	Procedure	Number of surgeries					
			2011	2012	2013	2014	2015	2016
Other	Low	Localized intra‐abdominal abscess surgery	2526	2944	3231	3262	2942	2764
	Low	Exploratory laparotomy	5036	6852	7532	8271	8982	9629
	Med	Acute diffuse peritonitis surgery	7753	9177	10 447	12 085	13 030	13 981
	Med	Ventral hernia surgery	5053	6095	11 387	12 298	12 494	12 896
	Med	Diaphragm suture	183	218	246	213	257	253
	Med	Esophageal hiatus hernia surgery	511	602	725	757	800	842
	Med	Retroperitoneal tumor surgery	622	837	806	805	807	850
	Med	Abdominal wall/mesenteric/omental tumor resection	979	1398	1402	1509	1506	1707
	Med	Gastrointestinal perforation closure	504	576	522	589	587	549
	High	Diaphragmatic hiatus hernia surgery	51	80	65	65	60	52

### 3.2 Eight main operative procedures

**Figure 2:**
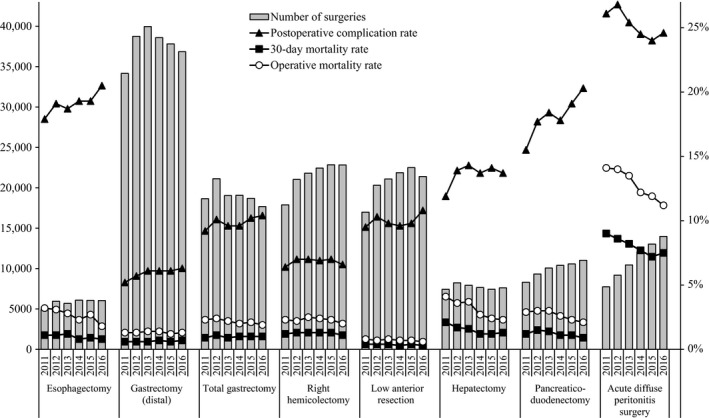
Annual changes of number of surgeries, 30‐day mortality, operative mortality, and complications: Analysis of eight major surgical procedures. Postoperative complication rate: the rate of Clavien‐Dindo (C‐D) classification grade III or higher complications.

The respective number of surgeries carried out annually for the eight main operative procedures, mortalities and complications between 2011 and 2016 are shown in Table 13 and Figure 2. Subsequently, the male : female ratio leaned toward males for all operative methods, with males particularly predominant with esophagectomy, gastrectomy (distal and total), and hepatectomy. In addition, the percentage of those patients who were ≥80 years was high for gastrectomy (distal and total), right hemicolectomy, and acute diffuse peritonitis surgery (Table 13). Regarding the institution groups in which surgeries were carried out, more than 70% of the surgeries were done at certified institutions and was particularly high for esophagectomy (94.5% in 2016), hepatectomy (non‐lateral segments) (90.7%), and pancreaticoduodenectomy (89.4%). Percentage of anesthesiologist participation was more than 90% for all eight procedures. Approximately 90% of esophagectomy, hepatectomy (non‐lateral segments), and pancreaticoduodenectomy procedures involved board‐certified surgeon participation, while the percentages of the same for right hemicolectomy and acute diffuse peritonitis surgery were 74.2% and 66.8% in 2016, respectively (Table 14). Table 15 shows the mortality rates of the eight main operative procedures. Other than acute diffuse peritonitis surgery, the postoperative 30‐day mortality rate was 0.3%‐2.1% and the operative mortality rate was 0.6%‐4.1%. The postoperative 30‐day mortality rate and operative mortality rate for acute diffuse peritonitis surgery was 7.5% and 11.2% in 2016, respectively (Table 15; Figure 2). Number of cases of acute diffuse peritonitis surgery is increasing; however, the morbidity and mortality rates are decreasing. There were differences in the incidence of complications according to organ site and procedure. Remarkably, mortality rates of low anterior resection were very low (0.3% and 0.6% for 30‐day mortality and operative mortality in 2016, respectively), and those of hepatectomy (1.3% and 2.3% in 2016) and acute diffuse peritonitis surgery (7.5% and 11.2% in 2016) have been gradually decreasing. Although the complication rates were gradually increasing for esophagectomy (20.5% in 2016) and pancreaticoduodenectomy (20.3% in 2016), the mortality rates for these procedures (0.8% and 1.8%, and 0.9% and 2.1% for 30‐day mortality and operative mortality in 2016, respectively) were decreasing.

**Table 13:** Annual changes of surgeries by sex, age group, and organ for the eight main operative procedures

Organ	Year	No. surgeries	Percentage by sex		Percentage according to age group (years)					
			Male	Female	<60	60 to <65	65 to <70	70 to <75	75 to <80	≥80
Esophagectomy	2011	4916	84.1	15.9	20.4	20.8	22.5	19.4	12.2	4.7
	2012	5946	84.4	15.6	19.7	21.3	20.7	20.3	13.1	4.9
	2013	5694	83.6	16.4	18.3	18.3	22.6	21.3	13.8	5.8
	2014	6091	84.0	16.0	18.7	17.8	22.8	22.0	13.4	5.2
	2015	6060	82.9	17.1	17.9	16.3	23.6	23.5	13.1	5.7
	2016	6041	81.7	18.3	17.8	15.8	25.3	21.6	14.3	5.2
Gastrectomy (distal)	2011	34 160	66.6	33.4	18.1	15.0	14.2	17.4	16.8	18.5
	2012	38 750	66.9	33.1	16.9	14.8	15.0	17.8	16.5	18.8
	2013	39 957	66.7	33.3	16.3	13.5	15.8	17.8	17.6	19.0
	2014	38 584	66.4	33.6	15.7	12.4	16.6	18.4	17.3	19.5
	2015	37 819	66.6	33.4	14.8	11.3	17.5	18.2	17.5	20.6
	2016	36 852	66.6	33.4	14.5	10.4	18.5	17.6	17.4	21.6
Total gastrectomy	2011	18 652	73.7	26.3	16.6	14.7	16.0	19.7	18.0	15.0
	2012	21 122	74.2	25.8	15.5	14.8	15.7	19.2	18.5	16.3
	2013	19 035	74.0	26.0	14.7	13.5	16.9	19.4	19.2	16.3
	2014	19 071	73.7	26.3	14.0	12.3	17.2	20.1	18.9	17.5
	2015	18 695	74.5	25.5	13.7	11.1	18.9	20.8	18.2	17.4
	2016	17 670	74.4	25.6	12.6	10.3	19.6	19.5	19.0	19.0
Right hemicolectomy	2011	17 890	50.5	49.5	12.8	11.6	13.1	17.3	18.8	26.5
	2012	21 034	50.3	49.7	13.1	10.9	13.1	17.0	19.0	26.9
	2013	21 814	50.6	49.4	13.0	10.0	13.4	17.6	18.9	27.1
	2014	22 446	50.6	49.4	12.0	9.2	13.8	18.2	18.6	28.2
	2015	22 850	50.5	49.5	11.5	8.6	14.6	18.1	18.1	29.1
	2016	22 829	51.3	48.7	11.4	7.7	15.9	16.7	18.5	29.8
Low anterior resection	2011	16 984	64.8	35.2	24.1	18.5	16.5	16.2	12.9	11.7
	2012	20 321	64.8	35.2	24.2	17.6	16.5	16.6	13.1	12.0
	2013	21 096	64.2	35.8	23.8	16.5	17.4	16.9	13.5	11.8
	2014	21 861	64.8	35.2	23.1	15.7	18.3	17.9	13.1	11.9
	2015	22 493	64.4	35.6	23.5	14.2	19.6	17.1	13.6	12.0
	2016	21 387	64.4	35.6	23.4	13.6	20.7	16.8	13.2	12.2
Hepatectomy (non‐lateral segments)	2011	7434	70.4	29.6	20.1	16.4	16.5	20.4	18.0	8.7
	2012	8239	69.5	30.5	19.8	16.1	17.4	19.5	18.5	8.8
	2013	7937	69.4	30.6	19.4	14.2	18.0	20.3	18.2	9.9
	2014	7666	69.2	30.8	18.5	13.8	18.5	21.5	17.6	10.0
	2015	7439	68.9	31.1	18.7	12.5	19.3	20.9	17.6	11.1
	2016	7610	68.7	31.3	18.0	11.9	21.1	20.4	17.5	11.1
Pancreaticoduodenectomy	2011	8305	61.9	38.1	16.1	16.0	17.3	20.9	18.8	10.9
	2012	9329	62.0	38.0	14.7	15.8	18.0	20.6	20.2	10.6
	2013	10 068	60.9	39.1	14.0	12.6	19.6	22.5	19.4	11.8
	2014	10 400	59.5	40.5	18.4	12.4	19.0	21.0	18.2	11.1
	2015	10 576	60.7	39.3	14.2	11.7	20.0	22.9	19.3	12.0
	2016	11 028	61.1	38.9	14.2	10.3	20.6	21.8	20.3	12.7
Acute diffuse peritonitis surgery	2011	7753	60.0	40.0	31.4	11.2	9.7	11.7	13.2	22.9
	2012	9177	61.0	39.0	30.3	11.2	10.1	11.6	13.4	23.4
	2013	10 447	60.1	39.9	29.1	10.3	11.5	11.8	13.1	24.1
	2014	12 085	61.2	38.8	28.4	9.5	12.2	12.3	12.9	24.7
	2015	13 030	59.4	40.6	28.2	8.9	12.5	13.1	12.3	25.0
	2016	13 981	60.2	39.8	27.4	8.6	13.4	12.4	12.3	26.0

**Table 14:** Institution and anesthesiologist and specialist participation rates by organ for the eight main operative procedures

Organ	Year	No. surgeries	Percentage by institution group			Anesthesiologist participation (%)	Board‐certified surgeon participation (%)	Medical practitioners (%)	
			Certified institution	Related institution	Other			Board‐certified surgeons	Non‐board‐certified surgeons
Esophagectomy	2011	4916	94.2	5.3	0.5	97.6	88.4	63.5	36.5
	2012	5946	78.3	4.9	16.8	98.1	89.0	64.8	35.2
	2013	5694	92.9	5.9	1.2	98.0	90.8	66.6	33.4
	2014	6091	93.6	4.7	1.7	98.6	92.6	70.2	29.8
	2015	6060	93.6	4.6	1.8	98.5	93.5	72.1	27.9
	2016	6041	94.5	3.8	1.7	98.8	93.7	73.2	26.8
Gastrectomy (distal)	2011	34 160	81.1	16.6	2.3	93.2	71.3	37.0	63.0
	2012	38 750	64.5	15.2	20.3	93.9	72.5	37.9	62.1
	2013	39 957	76.6	19.2	4.1	93.6	76.1	40.6	59.4
	2014	38 584	77.7	17.8	4.5	94.0	78.4	42.1	57.9
	2015	37 819	77.3	18.3	4.4	94.1	78.1	41.3	58.7
	2016	36 852	80.2	15.9	4.0	95.0	81.8	43.8	56.2
Total gastrectomy	2011	18 652	80.9	16.8	2.3	93.9	71.6	37.4	62.6
	2012	21 122	63.0	15.3	21.7	94.3	72.1	38.0	62.0
	2013	19 035	77.2	18.9	3.9	94.2	75.0	39.5	60.5
	2014	19 071	77.8	17.9	4.3	94.4	77.7	41.7	58.3
	2015	18 695	77.9	17.9	4.1	94.5	78.2	42.6	57.4
	2016	17 670	80.0	15.9	4.0	95.0	81.4	45.0	55.0
Right hemicolectomy	2011	17 890	75.7	21.2	3.1	92.7	66.0	30.5	69.5
	2012	21 034	60.0	18.3	21.7	93.0	67.1	30.8	69.2
	2013	21 814	72.1	22.3	5.6	92.9	69.7	32.6	67.4
	2014	22 446	71.2	23.1	5.7	93.4	71.9	33.6	66.4
	2015	22 850	72.1	22.0	5.9	94.1	72.4	33.5	66.5
	2016	22 829	73.8	20.1	6.1	94.5	74.2	34.3	65.7
Low anterior resection	2011	16 984	79.4	17.7	2.9	93.4	72.7	41.6	58.4
	2012	20 321	64.0	16.2	19.7	93.8	73.0	42.3	57.7
	2013	21 096	76.3	19.5	4.2	93.7	75.5	44.3	55.7
	2014	21 861	76.2	19.0	4.9	94.4	78.2	47.2	52.8
	2015	22 493	76.9	18.3	4.8	94.6	79.2	47.7	52.3
	2016	21 387	79.0	16.4	4.7	95.0	81.0	48.8	51.2
Hepatectomy (non‐lateral segments)	2011	7434	91.1	8.0	0.8	96.4	88.9	61.5	38.5
	2012	8239	75.9	7.9	16.3	96.8	89.3	64.0	36.0
	2013	7937	88.1	9.7	2.2	96.9	91.0	65.2	34.8
	2014	7666	88.2	8.7	3.1	96.7	92.3	66.6	33.4
	2015	7439	89.2	8.6	2.2	97.2	92.3	66.6	33.4
	2016	7610	90.7	7.1	2.1	97.1	93.3	67.7	32.3
Pancreaticoduodenectomy	2011	8305	87.8	11.0	1.2	95.9	85.7	58.7	41.3
	2012	9329	72.4	8.8	18.8	96.6	87.2	60.9	39.1
	2013	10 068	85.9	11.7	2.4	96.0	87.9	60.5	39.5
	2014	10 400	86.4	10.4	3.3	96.4	90.3	62.2	37.8
	2015	10 576	88.5	9.2	2.4	96.9	90.9	62.1	37.9
	2016	11 028	89.4	8.3	2.3	97.1	91.7	63.3	36.7
Acute diffuse peritonitis surgery	2011	7753	80.6	16.9	2.4	90.0	58.5	23.5	76.5
	2012	9177	65.2	16.4	18.4	90.4	59.4	22.7	77.3
	2013	10 447	77.7	18.1	4.2	91.2	62.4	23.9	76.1
	2014	12 085	77.7	17.2	5.1	91.9	63.3	25.1	74.9
	2015	13 030	79.8	15.9	4.3	92.2	64.5	24.9	75.1
	2016	13 981	82.2	13.8	4.0	93.0	66.8	26.1	73.9

**Table 15:** No. surgeries and mortality rates according to organ treated using the eight main operative procedures

Organ	Year	No. surgeries	No. postoperative complications^a^/rate (%)	No. 30‐day mortalities/rate (%)	No. operative mortalities/rate (%)
Esophagectomy	2011	4916	879/17.9	55/1.1	158/3.2
	2012	5946	1135/19.1	63/1.1	183/3.1
	2013	5694	1067/18.7	67/1.2	161/2.8
	2014	6091	1178/19.3	49/0.8	140/2.3
	2015	6060	1171/19.3	57/0.9	166/2.7
	2016	6041	1240/20.5	49/0.8	109/1.8
Gastrectomy (distal)	2011	34 160	1774/5.2	208/0.6	451/1.3
	2012	38 750	2205/5.7	232/0.6	516/1.3
	2013	39 957	2450/6.1	239/0.6	542/1.4
	2014	38 584	2356/6.1	264/0.7	523/1.4
	2015	37 819	2325/6.1	222/0.6	452/1.2
	2016	36 852	2314/6.3	249/0.7	473/1.3
Total gastrectomy	2011	18 652	1716/9.2	177/0.9	427/2.3
	2012	21 122	2135/10.1	224/1.1	503/2.4
	2013	19 035	1831/9.6	169/0.9	428/2.2
	2014	19 071	1840/9.6	185/1.0	379/2.0
	2015	18 695	1907/10.2	178/1.0	387/2.1
	2016	17 670	1835/10.4	174/1.0	336/1.9
Right hemicolectomy	2011	17 890	1150/6.4	213/1.2	410/2.3
	2012	21 034	1470/7.0	263/1.3	471/2.2
	2013	21 814	1527/7.0	280/1.3	538/2.5
	2014	22 446	1544/6.9	287/1.3	530/2.4
	2015	22 850	1607/7.0	301/1.3	534/2.3
	2016	22 829	1510/6.6	253/1.1	449/2.0
Low anterior resection	2011	16 984	1616/9.5	75/0.4	136/0.8
	2012	20 321	2092/10.3	88/0.4	149/0.7
	2013	21 096	2059/9.8	80/0.4	175/0.8
	2014	21 861	2098/9.6	70/0.3	152/0.7
	2015	22 493	2210/9.8	95/0.4	156/0.7
	2016	21 387	2306/10.8	68/0.3	126/0.6
Hepatectomy (non‐lateral segments)	2011	7434	886/11.9	155/2.1	303/4.1
	2012	8239	1146/13.9	142/1.7	293/3.6
	2013	7937	1135/14.3	130/1.6	290/3.7
	2014	7666	1052/13.7	94/1.2	208/2.7
	2015	7439	1049/14.1	87/1.2	182/2.4
	2016	7610	1046/13.7	96/1.3	178/2.3
Pancreaticoduodenectomy	2011	8305	1285/15.5	97/1.2	238/2.9
	2012	9329	1654/17.7	137/1.5	281/3.0
	2013	10 068	1853/18.4	142/1.4	307/3.0
	2014	10 400	1847/17.8	111/1.1	267/2.6
	2015	10 576	2025/19.1	120/1.1	247/2.3
	2016	11 028	2242/20.3	98/0.9	232/2.1
Acute diffuse peritonitis surgery	2011	7753	2022/26.1	697/9.0	1096/14.1
	2012	9177	2456/26.8	785/8.6	1289/14.0
	2013	10 447	2652/25.4	861/8.2	1408/13.5
	2014	12 085	2966/24.5	927/7.7	1472/12.2
	2015	13 030	3126/24.0	943/7.2	1551/11.9
	2016	13 981	3445/24.6	1052/7.5	1572/11.2

a Complications were defined by Clavien‐Dindo grade IIIa‐V.

Increase in the incidence of endoscopic surgery is shown in Table 16. Endoscopic surgeries have been prevalent especially in gastrointestinal procedures, while laparoscopic hepatectomy or pancreaticoduodenectomy have been carried out in limited institutions. Even for acute diffuse peritonitis, laparoscopic surgery has been done in 15.5% of all surgeries in 2016.

**Table 16:** Annual changes of endoscopic surgeries for the eight main operative procedures

Organ	Year	No. surgeries	Endoscopic surgery	% Endoscopic surgery
Esophagectomy	2011	4917	1525	31.0
	2012	5948	2200	37.0
	2013	5694	2315	40.7
	2014	6091	2569	42.2
	2015	6060	2659	43.9
	2016	6041	2961	49.0
Gastrectomy (distal)	2011	34 198	10 801	31.6
	2012	38 774	13 098	33.8
	2013	39 959	16 507	41.3
	2014	38 584	14 432	37.4
	2015	37 819	14 357	38.0
	2016	36 852	15 333	41.6
Total gastrectomy	2011	18 674	2258	12.1
	2012	21 139	3060	14.5
	2013	19 038	3669	19.3
	2014	19 071	3620	19.0
	2015	18 695	3707	19.8
	2016	17 670	4007	22.7
Right hemicolectomy	2011	17 899	4842	27.1
	2012	21 047	6954	33.0
	2013	21 816	9124	41.8
	2014	22 446	8269	36.8
	2015	22 850	8755	38.3
	2016	22 829	9622	42.1
Low anterior resection	2011	16 996	5018	29.5
	2012	20 333	7649	37.6
	2013	21 098	10 814	51.3
	2014	21 861	11 298	51.7
	2015	22 493	12 080	53.7
	2016	21 387	12 478	58.3
Hepatectomy (non‐lateral segments)	2011	7440	242	3.3
	2012	8246	389	4.7
	2013	7938	567	7.1
	2014	7666	392	5.1
	2015	7439	127	1.7
	2016	7610	433	5.7
Pancreaticoduodenectomy	2011	8310	67	0.8
	2012	9340	121	1.3
	2013	10 069	156	1.5
	2014	10 400	124	1.2
	2015	10 576	53	0.5
	2016	11 028	118	1.1
Acute diffuse peritonitis surgery	2011	7767	488	6.3
	2012	9189	652	7.1
	2013	10 452	1070	10.2
	2014	12 085	1381	11.4
	2015	13 030	1638	12.6
	2016	13 981	2164	15.5

## 4 DISCUSSION

Since the start of NCD registration in 2011, surgeons in Japan, especially JSGS members, have constructed a robust nationwide database. We can see the real clinical status of surgical outcomes in Japan. The number of registered surgeries has been increasing year by year. Mortality rates for all of the procedures seem to be acceptable as a nationwide outcome, as they are satisfactorily lower than those reported from other countries.^24^, ^25^ These results may be explained by the high participation rate of board‐certified surgeons. Board‐certified surgeons in gastroenterological surgery contribute to favorable outcomes in Japan.^21^ A multivariable logistic regression model showed that a greater board‐certified surgeon number in hospitals predicted a favorable surgical outcome in relation to operative mortality. Analyzing the data of NCD, we can validate an appropriate number of board‐certified surgeons required to authorize hospitals to carry out invasive surgeries. On the basis of this report, we are now planning to make a revised risk model using the recent data. In the field of hepato‐biliary‐pancreatic surgery, The Japanese Society of Hepato‐Biliary‐Pancreatic Surgery (JSHBPS) established a board‐certification system for expert surgeons (hepato‐biliary‐pancreatic [HBP] field) in 2008, and certification of expert surgeons started in 2011.^26^, ^27^ Miura et al.^28^, ^29^ reported that a multiple logistic regression model showed that the cut‐offs of high‐level HBP surgeries carried out per year at hospitals that predicted 30‐day mortality after hepatectomy of more than one segment and pancreatoduodenectomy were 10 and 50. Competencies and requirements for board‐certified institutions, instructors, and expert surgeons to carry out hepatectomy or pancreatoduodenectomy were found to be appropriate.

As for complications, this is the first report of the annual complication rate in the 115 selected gastrointestinal operative procedures in the training curriculum for board‐certified surgeons in gastroenterology, and eight main procedures representing the performance of surgery using NCD data. There were differences in the incidence of complications according to organ site or operative procedure. As the complication rates in this report were the sum of all complications with C‐D classification grade III or higher, it is necessary to further analyze on postoperative complication. The registered number of surgeries has been increasing, and older are patients increasing, the trend of which generally means difficulty in maintaining a low complication rate. However, mortality rates have been maintained at a rather low level. Strict indication for surgery and appropriate perioperative management might affect the low mortality rate. It has been shown that performance data released to the public promote quality improvement activity at the hospital level,^30^, ^31^ and vice versa.^32^ It is necessary to analyze with explanations the tendency of surgical outcomes over time. A risk‐adjusted analysis based on nationwide data allows personnel to establish and provide feedback on the risks that patients face before undergoing a procedure.^11^ The NCD also provides data on each facility's severity‐adjusted clinical performance (benchmark), which can be compared with national data. We can trace periodically where we are in the national standard.

Thinking of the future development of NCD, long‐term clinical outcomes will be demanded, especially in cancer registries. The NCD generalizes site‐specific cancer registries by taking advantage of their excellent organizing ability. Some site‐specific cancer registries, including pancreatic, breast, and liver cancer registries have already been combined with the NCD.^33^ Aggregation of the cancer registration system and NCD would definitely produce a novel and important database, not only in the field of clinical medicine but also public health. Another possible linkage to NCD is the medical insurance database including diagnosis procedure combination (DPC) data, which includes not only clinical information on disease but also the medical costs by disease or treatment.^34^

After the first stage of the establishment of the national database, NCD has been proceeding to the second stage, development and utilization. Many studies are in progress to improve quality control of surgical procedures using the NCD. Future evolution of the NCD will be promising with impacts to the public.

## DISCLOSURE

Conflicts of Interest: Authors declare no conflicts of interest concerning this project.
